# Effect of CaCl_2_ Sprays in Different Fruit Development Stages on Grape Berry Cracking

**DOI:** 10.3389/fpls.2022.870959

**Published:** 2022-06-13

**Authors:** Hao Shi, Xiangyu Zhou, Mengling Qin, Wenlong Wang, Xiaoe He, Wenhua Zhou

**Affiliations:** ^1^College of Food Science and Engineering, Central South University of Forestry and Technology, Changsha, China; ^2^College of Agriculture and Forestry Science, Hunan Applied Technology University, Changde, China; ^3^School of Food Science and Nutrition, University of Leeds, Leeds, United Kingdom

**Keywords:** fruit cracking, fruit development stages, calcium treatment, grape peel, abscisic acid

## Abstract

Grape cracking is a common phenomenon that can reach more than 50% in some varieties and can cause enormous economic losses. “Li Xiu” grapes in different developmental stages were treated with calcium chloride (5 g/L), and the cracking rate and related biochemical and genetic indices were measured in the fruit ripening stage. The results showed that calcium treatment during the flowering period could significantly reduce grape cracking. Based on the experimental results, there are several reasons as follows: first, calcium spraying during the flowering period was more favorable to calcium absorption in grapes, and the increased calcium content in the peels helped to improve the mechanical properties of the peels, thus increasing crack resistance; second, calcium treatment reduced the expression levels of genes related to glucose metabolism, which in turn reduced PG and cellulase activities, delaying the degradation of pectin and cellulose and resulting in more structural integrity of the peels; third, calcium treatment increased fruit hardness and reduced fruit ventral pressure by decreasing the expression levels of ABA-related genes and synthesis of endogenous abscisic acid (ABA), soluble sugars (SSs), and total soluble solids (TSSs).

## Introduction

Grapes are one of the most popular fruit crops in the world. They are a rich source of nutrients (minerals, active polysaccharides, anthocyanins, resveratrol, vitamins, etc.). However, some grape varieties are prone to cracking, which leads to fruit decay. Grape cracking is currently a serious problem in vineyards around the world (Yamamura and Naito, 1985; Inchang et al., 2007; Zofoli et al., 2008). Fruit cracking can lead to poor appearance, reduced quality and shelf life, susceptibility to fungal infections, and, ultimately, unmarketable fruits. Some grape varieties that are prone to cracking can have cracking rates of more than 90% at maturity, which causes significant commercial losses for grape growers. The “Li Xiu” grape is an American variety with large and thick leaves, disease resistance, early maturity, consistent yield, no kernels, high quality, high sugar content, and intense aroma. However, it is very prone to fruit cracking in southern China (because of hot and rainy climate).

There are many causes of fruit cracking. According to relevant studies, management practices, environment, genetic factors, physiology, and biochemistry are all associated with fruit cracking (Sofia et al., 2018; Kaur et al., 2019). The composition and structure of the pericarp cell wall will affect the mechanical properties of the pericarp, which is an important factor in determining whether the grape is prone to cracking (Yang et al., 2016; Brüggenwirth and Knoche, 2017; Jiang et al., 2019). The pericarp cell wall contains mainly minerals, cellulose, hemicellulose, starch, and pectin. The content of these substances in the pericarp is directly related to fruit cracking. Furthermore, the activity of cellulase and pectinase in the fruit is closely related to fruit cracking. After fruit ripening, cellulase and pectinase activities increase, leading to degradation of polysaccharides in the cell wall, which reduces the mechanical properties of the pericarp and makes it more prone to cracking (Cantu et al., 2008).

Many studies have shown that soluble sugar content and soluble solid content also have a relationship with fruit cracking. For example, Yu et al. (2020) confirmed that nordihydroguaiaretic acid treatment of grapes reduced ABA, SS, and TSS content in grapes. Furthermore, Chen et al. (2019) demonstrated that ethylene accelerated the degradation of starch, inhibited the synthesis of protopectin, and increased the level of soluble pectin, which is consistent with the phenotype of ethylene-induced fruit cracking. More importantly, the increased sugar content in the fruit reduced water potential, thereby increasing the water uptake and movement of the pericarp and increased fruit cracking.

Hormones also affect the cracking of fruits to a large extent (Lu et al., 2017). For example, Jáuregui-Riquelme et al. (2017) demonstrated that the application of CPPU in the pre-anthesis stage altered grape development and improved berry size and berry potential tolerance to post-harvest cracking and spoilage. Josan et al. (1995) confirmed that the lemon had the lowest fruit-cracking rate with 40 ppm NAA, followed by 20 ppm NAA and 10 ppm GA3. Moreover, Wang et al. (2019) demonstrated that the litchi fruit cracking-susceptible cultivar “Baitangying” pericarp had higher abscisic acid contents, increased biosynthesis of ethylene and jasmonic acid, and reduced biosynthesis of auxin and brassinosteroid.

The mineral nutritional status of fruits is closely related to fruit cracking. Tonetto de Freitas and Cai-Zhong JiangMitcham (2012) confirmed that blossom-end rot was recognized to be caused by calcium (Ca) deficiency. Brown et al. (1995) confirmed that copper hydroxide alone, at low concentrations or in combination with calcium hydroxide, significantly reduced fruit cracking. Correia et al. (2020) confirmed that calcium (Ca) and gibberellic acid (GA) sprays were demonstrated to be the best compounds for increasing yield and reducing cherry cracking as well as improving photosynthetic performance and leaf metabolite content. Ca is an essential element for plants and plays many important roles in healthy growth of plants. Ca is involved in the structure of cell walls and cell membranes, and it has a certain effect on cell development and signal transduction (White and Broadley, 2003; Hashimoto and Kudla, 2011; Ranty et al., 2016).

Although the role of calcium in preventing fruit cracking has been studied, when the appropriate time for calcium treatment is and its underlying mechanisms have not been fully clarified (Simon, 2006). This study has four objectives: (1) to obtain the optimal treatment period by applying calcium treatments to grapes in different growth stages to provide new ideas for the prevention of fruit cracking, (2) to examine how calcium treatment reduces the cracking rate of grapes (“Li Xiu”) in terms of the apparent structure of the grape skin, (3) to elucidate the mechanisms underlying the physiological and molecular bases of calcium treatment in preventing fruit cracking, and (4) to reduce post-harvest cracking rates, improve fruit storability, and promote their economic value.

## Materials and Methods

### Plant Materials and Treatments

Seven-year-old “Li Xiu” grapes were used as experimental materials, cultivated under a rain shelter and in the teaching base of Hunan Applied University in Changde, Hunan, China (28°99′N, 111°67′E). Sprays were carried out with 5 g/L CaCl_2_ in the inflorescence (April 14th, as treatment I), flowering (May 7th, as treatment II), fruit development (May 28th, as treatment III), and end of fruit development (June 20th, as treatment IV) periods of fruit growth, with six grapevines per treatment. No calcium spraying was conducted on the control samples. When the fruits of each treatment group (I, II, III, IV, and control) were ripe (with purple-red skin), grape berries were collected randomly from each treatment sample. The grape berries were flash-frozen in liquid nitrogen and then stored at −80°C.

### Determination of Grape Fresh Weight, Berry Volume, and Cracking Rate

#### Experimental Samples From I, II, III, IV, and Control

Fresh fruit weight and berry volume were determined from 20 randomly selected fruits of each treatment, and the mean value was calculated. Fruit cracking rate was calculated according to the following formula: fruit-cracking rate (%) = number of cracked berries/total number of berries × 100. Fruit breaking force was determined with an ST-Z16 texture analyzer (Shengtai, Shandong, China). Fruit cracking index was calculated according to the following formula: fruit cracking


$$\begin{array}{l} \text{index }\left(\text{\%}\right)=\frac{{\left(\text{N}0^{*}0+N1^{*}1+N2^{*}2+N3^{*}3\right)}^{*}100}{\text{N}{\text{t}}^{*}3}\%. \end{array}$$

N0: normal fruit, N1: fruit cracks are not more than 1 cm, N2: fruit cracks are 1 to 2 cm, N3: fruit cracks more than 2 cm, Nt: total number of statistics.

### Determination of Grape-Related Physiological Indicators

#### Experimental Samples From I, II, III, IV, and Control

The (ABA) ELISA kit was used to determine the amount of endogenous ABA in grape fruit (Yingxin, Shanghai, China) described by Chen et al. (2006) and Sun et al. (2016), nanograms of ABA per grams of grape (ng. g^−1^ FW). The soluble sugar (SS) content in grapes was determined by the sulfuric acid-phenol method, grams of SS per hundred grams of grape (%). Determination of total soluble solids (TSSs) was conducted with a WYA-2S digital automatic Abbe refractometer (Inesa, Shanghai, China). The total anthocyanin content of grape skins was measured with a UV-1800 ultraviolet (Shimadzu, Kyoto, Japan) as described by Yu et al. (2020), milligrams of SS per grams of grape peel (mg kg^−1^ FW).

### Cellulose, Pectin Content, and Related Enzyme Activities

#### Experimental Samples From I, II, III, IV, and Control

Take 2 grams of grape peel in a mortar and grind it with liquid nitrogen, then add 10 ml of pre-cooled buffer, shake on ice for 3 min, frozen, and centrifuge (4°C), and the supernatant was the enzyme solution. The pectinase and cellulase activities of berry peels were measured with the DNS (3,5-dinitrosalicylic acid) colorimetric method. CMC-Na and polygalacturonic acid were used as substrates for determination of pectinase and cellulase, respectively, as described by Lohani et al. (2004).

Take 1 gram of grape peel in a mortar and grind it with liquid nitrogen, then add 10 ml of 95% ethanol and boil it with water for 30 min. After centrifugation, 10 ml of absolute ethanol, chloroform-methanol (1:1, *V/V*), and acetone were added in sequence to remove impurities. After centrifugation, we added 10 ml of deionized water to the residue, which was shaken in a shaker for 12 h, and the supernatant was water-soluble pectin (WSP). After centrifugation, we added 10 ml of 66% sulfuric acid to the residue, which was shaken in a shaker for 1 h, and the supernatant was cellulose. Cellulose content was determined with the anthrone-sulfuric acid method. The content of water-soluble pectin was determined with the m-hydroxybiphenyl method (Deng et al., 2005).

### RNA Extraction and q-PCR Assay

#### Experimental Samples From I, II, III, IV, and Control

The total RNA of grape peels was extracted with a Trizol kit (Yuanye, Shanghai, China). RNA was reverse-transcribed into cDNA. The sequences are listed in Table 1. qRT-PCR primers were synthesized in a Sangon Bioengineering Co., Ltd. (Shanghai, China) RT-PCR reaction system: 1 μl template, 10 μl SYBR Green qPCR SuperMix, 1 μl upstream primer (10 nM), 1 μl downstream primer (10 nM), supplemented with double distilled water to 20 μl. Reaction conditions were as follows: pre-denaturation at 95°C for 5 min, denaturation at 95°C for 10 s, annealing at 60°C for 30 s, and fluorescence detection for 15 s, 40 cycles. A relative quantitative 2^ΔΔCt^ analysis (CT value comparison method) was performed using β-actin as an internal reference to calculate the relative expression of each gene in the samples (Sun et al., 2010).

**Table 1:** Details of the qRT-PCR primers.

**Genes**	**Forward primer (5^**′**^to 3^**′**^)**	**Reverse primer (5^**′**^to 3^**′**^)**	**References**
VvNCED1	GGTGGTGAGCCTCTGTTCCT	CTGTAAATTCGTGGCGTTCACT	Sun et al. (2010)
VvCYP707A1	GAAACATTCACCACAGTCCAGA	AGCAAAAGGGCCATACTGAATA	Sun et al. (2010)
VvBG1	GGCTTCCTACTCCATCTTTC	GCAGTTTGGTGACAGGGTGA	Sun et al. (2015)
VvSuS	CTGGGGTTTATGGGTTCTG	AATGCCTCTGCCTTTTAGC	Lin et al. (2018)
VvHT3	GCTACTCTCTACTCGTCCGCTTTG	CCCGTCATTGCTTCCGAACTTG	Lecourieux et al. (2010)
VvGIN1	CCTTCTCCATCCCATCGTAACC	GGTTATCCAAGTTTCCAACCAACC	Lu et al. (2017)
VvUFGT	GGGATGGTAATGGCTGTGG	ACATGGGTGGAGAGTGAGTT	Jeong et al. (2004)
VvmybA1	TAGTCACCACTTCAAAAAGG	GAATGTGTTTGGGGTTTATC	Jeong et al. (2004)
VvDFR	GCATGGAAGTATGCCAAGGAAA	TCGGGGAAAGAGCAGTTATGAG	Zhang et al. (2019)
GAPDH	TATTAGGAACCCAGAGGAGATT	TCCTGTGGACAATGGATGGA	

### Scanning Electron Microscopy (SEM) and Transmission Electron Microscope (TEM) of Grape Peels

Grape peel samples were taken from the equator uniformly, the size was about 3 × 3 × 1 mm^3^. After grape skin treatment, the cross-section of grape peels was observed with a scanning electron microscope (SU8100, HITACHI, Japan) and a transmission electron microscope (HT7700, HITACHI, Japan), the content of mineral elements in the grape peels was analyzed with an X-ray energy spectrometer (AZtecLive Ultim Max 100: Oxford Instruments, England), and Image-Pro plus 6.0 (Media Cybernetics, Inc., Rockville, MD, United States) was used to determine cell wall thickness.

### Post-Harvest Grape Cracking Assay

Experimental samples were taken from the fruits of groups I, II, III, IV, and control at maturity. Fruits of uniform size and color and free from mechanical damage and diseases were taken. Fruit storage conditions were relative humidity: 95 ± 2% and temperature: 4 ± 0.5°C. Fruit cracking rate was measured every 12 days for each group, for a total of 5 times. All experiments were performed in triplicate.

### Statistical Analysis

All data were expressed as mean ± SD by measuring three independent replicates, and the measurements were performed using SPSS version 22.0 (SPSS Inc., IBM, Armonk, NY, United States) and Origin 9.0 (Origin Lab Corporation, United States). Significance was evaluated by one way analysis of variance (ANOVA). Tukey's multiple range test was performed following a significant test. Different Lowercase letters indicate highly significant differences between different concentrations (*P* < 0.05).

## Results

### Texture Characteristics and TSS Content

The fruit-cracking rate and cracking index of samples treated with CaCl_2_ were lower than those of the control samples (Table 2). Specifically, the fruit-cracking rate and cracking index were represented about four and 10 times lower (*P* ≤ 0.05) in the II sample than in the control group. Then, we found that CaCl_2_-treatment increased the fruit breaking force of the grapes (Table 2), the II sample was increased by 16.55% compared with the control group, while TSS content was decreased in the treatment group. Specifically, the II sample was decreased by 8.94% compared with the control group (Table 2). In addition, fresh fruit weight and berry volume were increased after calcium treatment (Table 2).

**Table 2:** Effects of the CaCl_2_ application on cracking, texture characteristics, and total soluble solids (TSS) content.

**Treatment**	**Cracking rate (%)**	**Cracking index (%)**	**Breaking force (g)**	**TSS content (%)**	**Fresh weight (g)**	**Berry volume (C × L)**
I	5.34 ± 0.70a	3.83 ± 0.17ab	569.27 ± 24.06b	13.83 ± 0.09b	6.95 ± 1.2	23.7 × 24.6
II	1.40 ± 1.07b	0.51 ± 0.36b	607.70 ± 6.74a	12.90 ± 0.36c	7.32 ± 1.5	24.2 × 25.5
III	3.24 ± 2.16ab	1.42 ± 1.51b	593.70 ± 7.71ab	13.53 ± 0.12b	7.20 ± 1.1	23.9 × 24.7
IV	3.52 ± 0.17ab	1.76 ± 0.41b	581.97 ± 10.16ab	13.23 ± 0.12Bc	6.97 ± 0.8	23.9 × 24.6
Control	6.84 ± 1.80a	5.69 ± 1.59a	521.40 ± 19.81c	14.17 ± 0.25a	6.73 ± 0.7	21.3 × 22.5

*Data are presented as mean ± standard. Lowercase letters represent significant differences (P ≤ 0.05). C × L represents cross × longitudinal*.

### ABA Content and Related Gene Expression Levels

**Figure 1:**
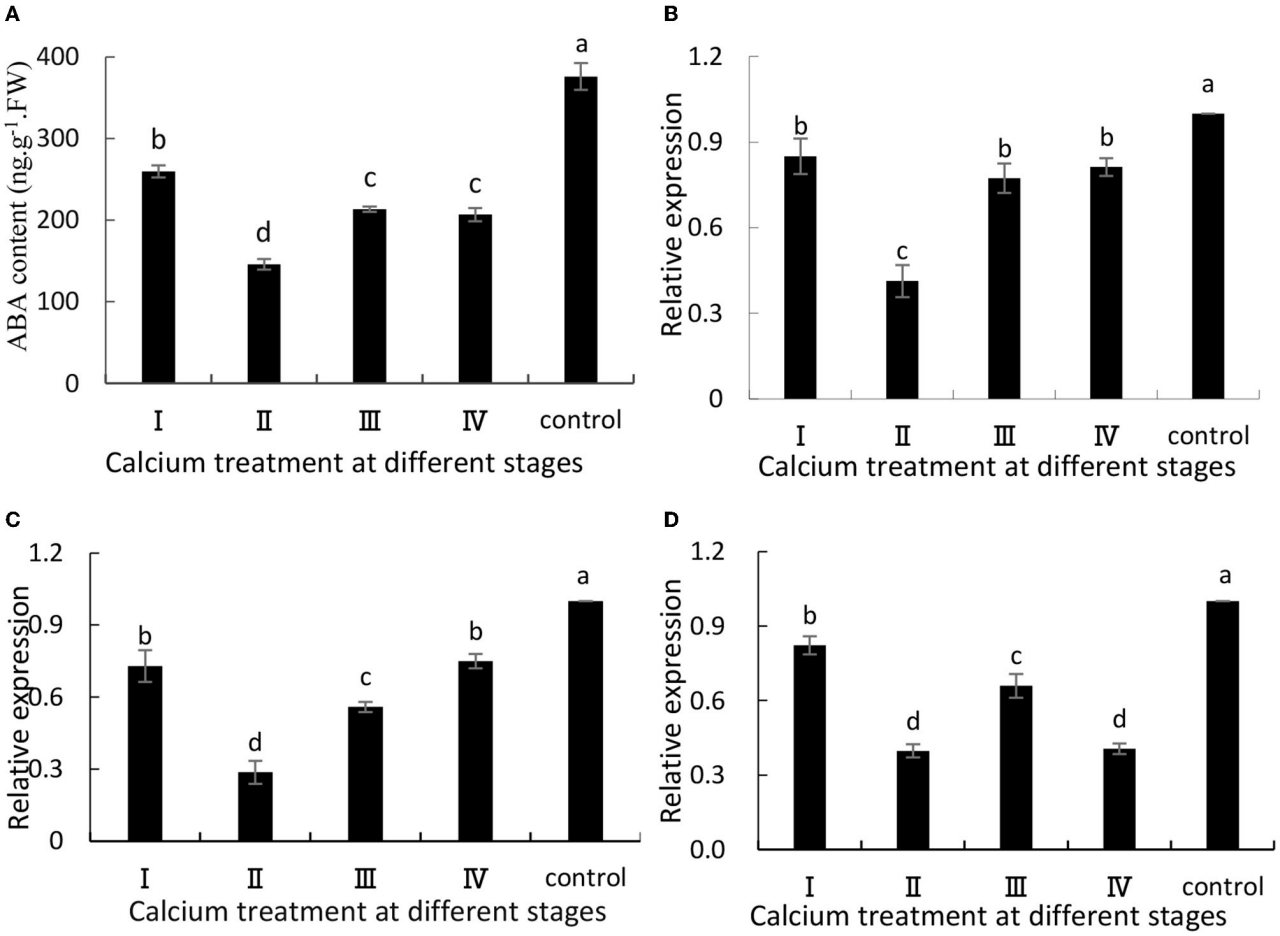
**(A)** ABA content, and expression of **(B)** VvNCED1, **(C)** VvCYP707A1, and **(D)** VvBG1. Data are presented as mean ± standard. Lowercase letters represent significant differences (*P* < 0.05).

When the grapes were treated with CaCl_2_, ABA synthesis was significantly (*P* ≤ 0.05) inhibited (Figure 1A). Specifically, the II sample was decreased by 61.26% compared with the control group. Additionally, VvNCED1 (Figure 1B) and VvBG1 (Figure 1D) are key genes for ABA synthesis, and their expression levels (*P* ≤ 0.05) were significantly down-regulated when CaCl_2_was applied. Specifically, the II sample was decreased by 59 and 60% compared with the control group. Moreover, the expression level of VvCYP707A1 related to ABA degradation was significantly (*P* < 0.05) decreased after CaCl_2_ treatment (Figure 1C), and the II sample was decreased by 71% compared with the control group.

#### Cellulose, Pectin Content and Related Enzyme Activities

**Figure 2:**
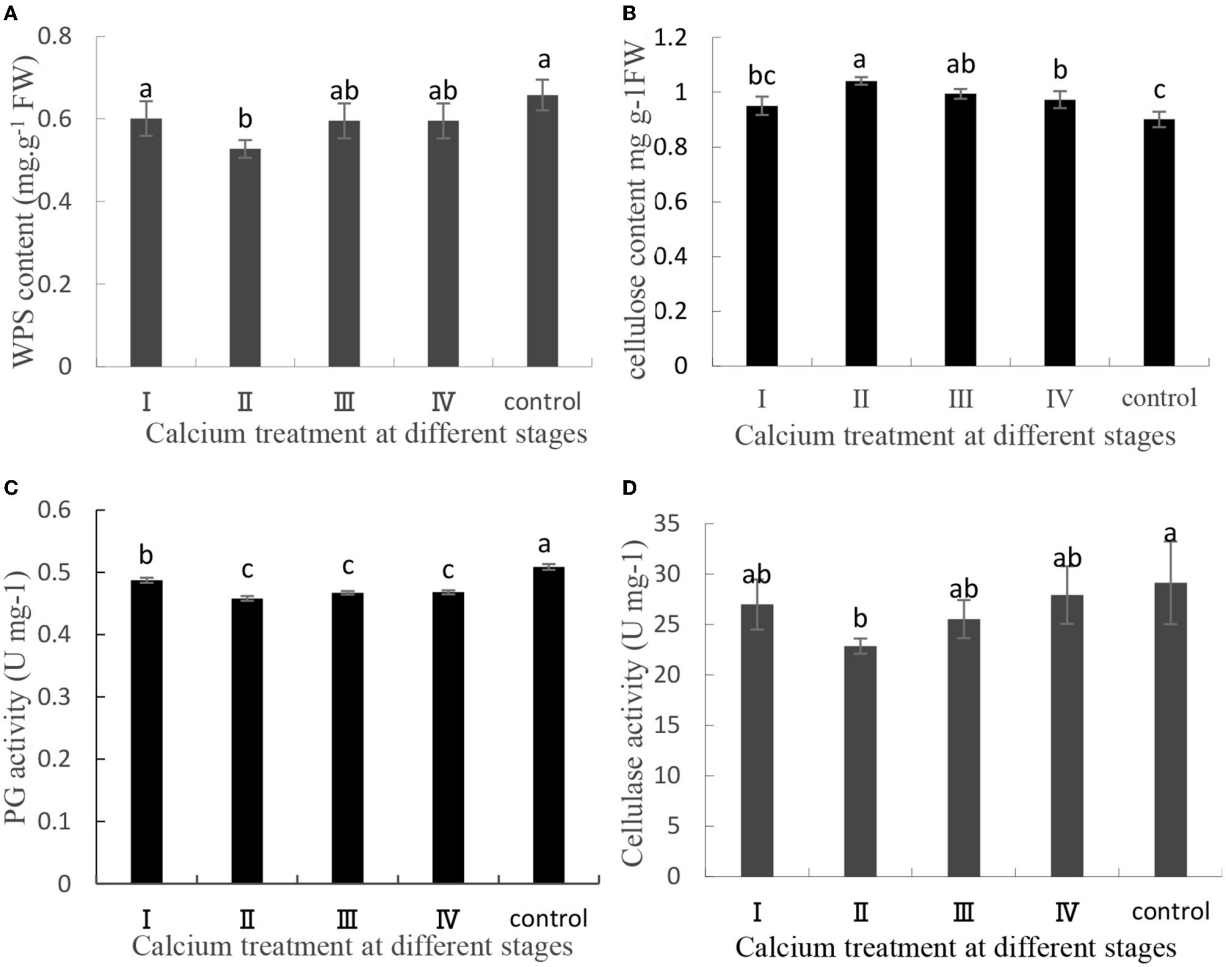
**(A)** Water-soluble pectin content, **(B)** cellulose content, **(C)** PG activity, and **(D)** cellulase activity contents of the grape berry peel. Data are presented as mean ± standard. Lowercase letters represent significant differences (*P* < 0.05).

The content of pectin, cellulose, and related enzyme activities in the cell wall of the pericarp is related to fruit cracking (Yu et al., 2019, 2020). In this study, pectin and cellulose were separated and quantified, and related enzyme activities were measured. In CaCl_2_-treated samples, the cellulose content was increased, whereas the WPS content, PG activity, and cellulase activity were decreased (Figure 2). Specifically, the WPS content in the II sample was decreased by 19.84% (Figure 2A), and the cellulose content in the II sample was increased by 7.7% (Figure 2B). Furthermore, the PG activity and cellulase activity in the II sample were decreased by 9, and 21.55%, respectively (Figures 2C,D). Thus, CaCl_2_ can decrease the PG activity and cellulase activity relative to the control level and then delay pectin and cellulose degradation.

### Soluble Sugar Content and Related Gene Expression Levels

**Figure 3:**
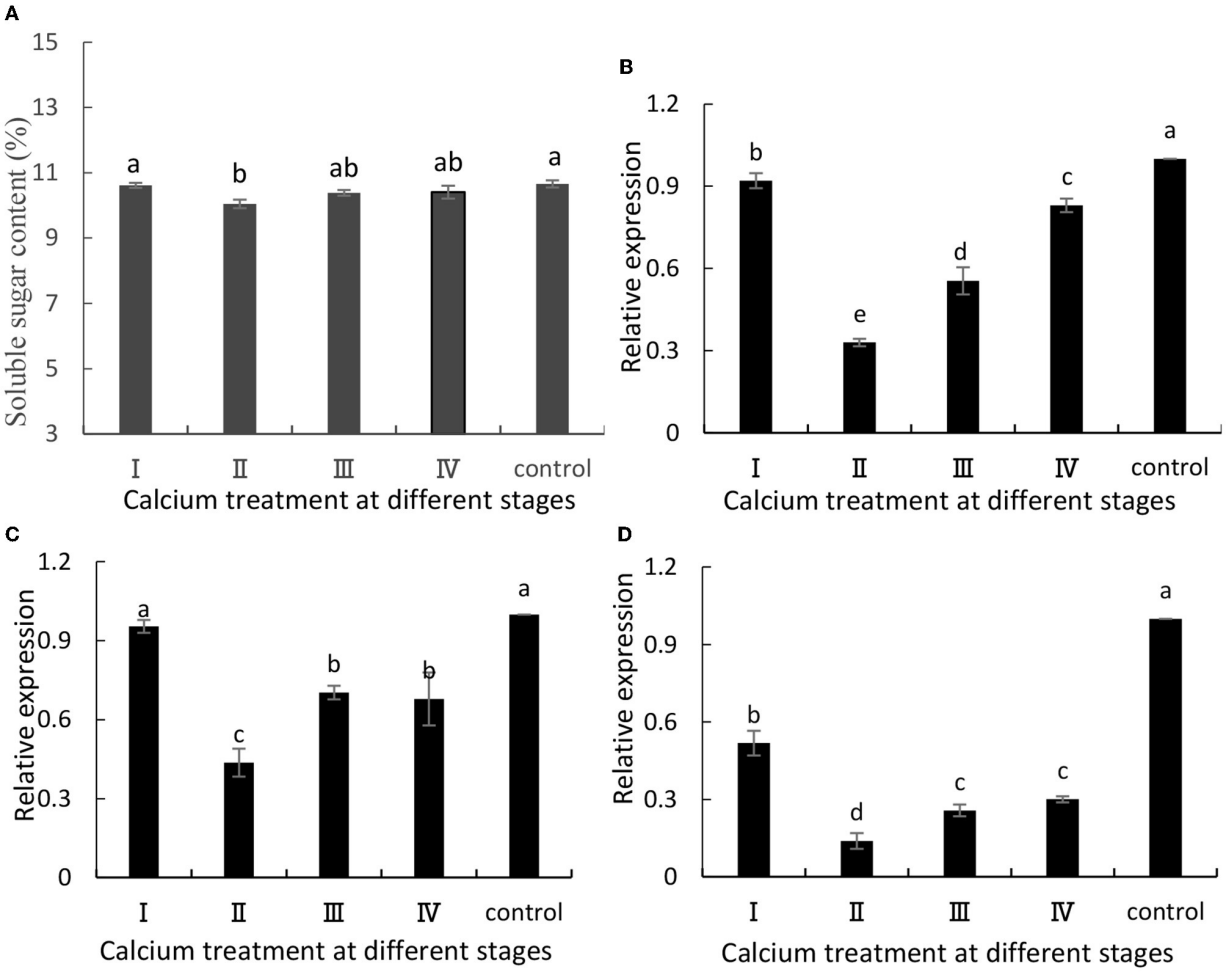
**(A)** Soluble sugar content and expression of **(B)** VvSuS, **(C)** VvHT3, and **(D)** VvGIN1. Data are presented as mean ± standard. Lowercase letters represent significant differences (*P* < 0.05).

The ABA content in fruits is related to fruit cracking (Lin et al., 2018). Metabolism of ABA would favor the accumulation of soluble sugars and TSSs in grapes. In this study, when grapes were ripe, we measured their soluble sugar content, and the content of soluble sugars was decreased in the calcium-treated group (Figure 3A). Specifically, the II sample was decreased by 5.8% compared with the control group. Furthermore, VvSUS (Figure 3B), VvHT3 (Figure 3C), and VvGIN1 (Figure 3D)] are crucial genes involved in sugar synthesis, and their expression levels were decreased. Specifically, the II sample was decreased by 67, 54, and 86%, respectively, compared with the control group (Figure 3).

### Total Anthocyanin Content and Related Gene Expression Levels

**Figure 4:**
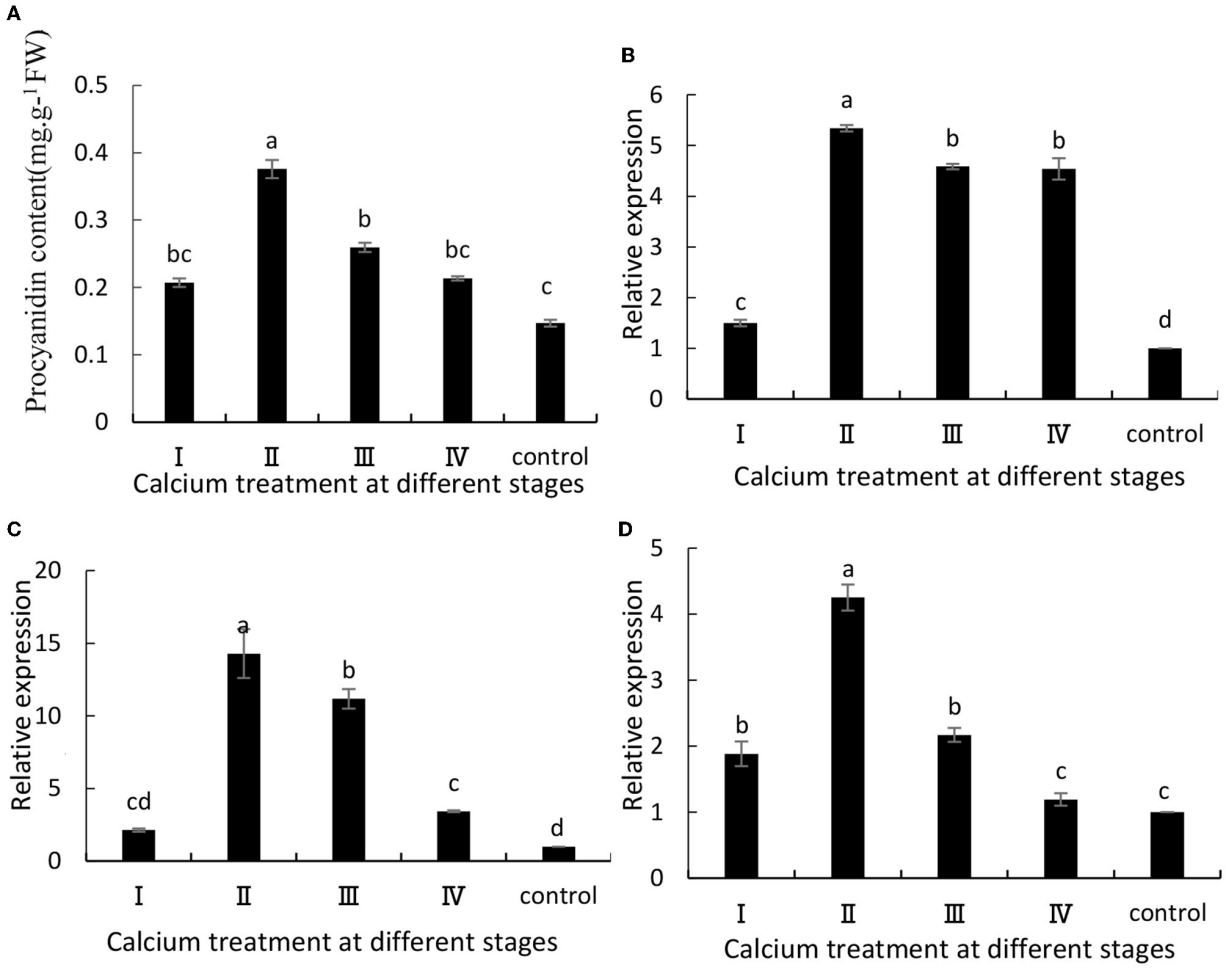
**(A)** Anthocyanin content and expression of **(B)** VvUFGT, **(C)** VvmybA1, and **(D)** VvDFR. Data are presented as mean ± standard. Lowercase letters represent significant differences (*P* < 0.05).

The anthocyanin content in grapes is related to ABA metabolism and fruit cracking (Darzi and Ahsan, 2010). In this study, when the grape berries were treated with calcium chloride, there was a significant increase in anthocyanin content in the grapes at maturity (Figure 4A). Specifically, the II sample was increased by 1.58 times compared with the control group. Furthermore, VvUFGT, VvmybA1, and VvDFR are key genes involved in anthocyanin synthesis, and their expression levels were significantly (*P* ≤ 0.05) higher in the CaCl2-treated samples than in the control group (Figures 4B–D). Specifically, the II sample was increased by 5.34, 14.3, and 4.25 times, respectively, compared with the control group (Figure 4).

### Effects of TheCaCl_2_ Application on the Microstructure of Grape Peels

#### Scanning Electron Microscopy Results

**Figure 5:**
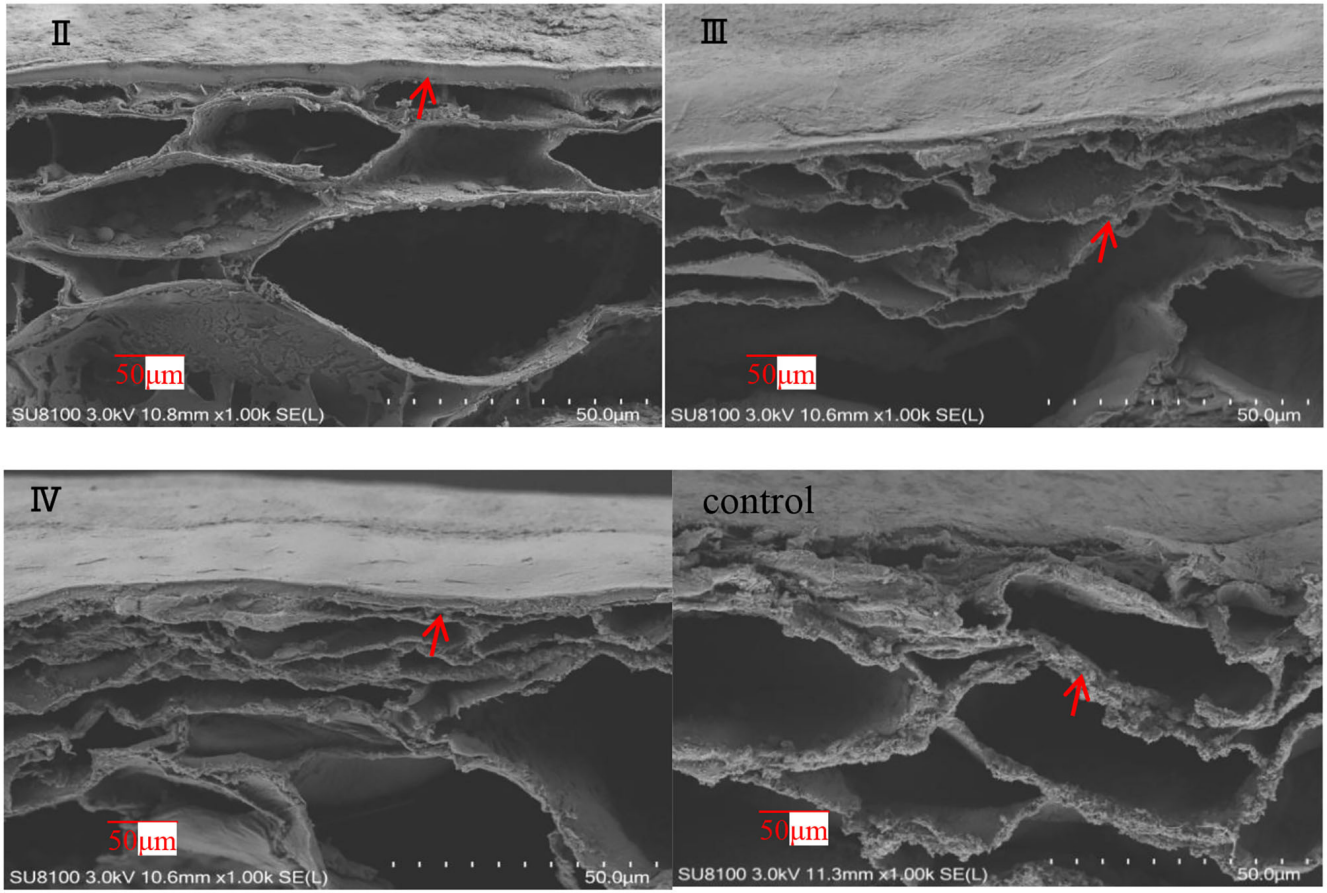
SEM of grape peels with calcium treatment in different stages (II represents ripening grape skins treated with calcium spray at flowering, III represents ripe grape skins treated with calcium spray during fruit development, and IV represents ripe grape skins treated with calcium spray at the end of fruit development) (grape peel, arrows).

The effects of calcium on the structure of grape peels are shown in Figure 5. Due to the poor effectiveness of (I), we did not conduct further studies on (I). The pericarp in the calcium treatment was smooth and flat. However, the pericarp of the control group was rough and wrinkled (Figure 5). Moreover, the calcium content of the berry skin in the calcium treatment was significantly higher compared with that of the control (Table 3). Specifically, the calcium content was represented about two times higher (*P* ≤ 0.05) in the II sample than in the control group. This study has shown that low Ca content in the pericarp is related to fruit cracking, and that spraying calcium can reduce fruit cracking by increasing Ca content in the pericarp (Table 3).

**Table 3:** Content of mineral elements in pericarp treated with calcium in different periods.

**Treatment**	**C/Wt%**	**O/Wt%**	**Na/Wt%**	**Mg/Wt%**	**Si/Wt%**	**Ca/Wt%**	**Zr/Wt%**
II	57.60 ± 0.18	41.02 ± 0.18	0.49 ± 0.02	0.10 ± 0.01	0.02 ± 0.01	0.62 ± 0.01	0.15 ± 0.08
III	61.5 ± 0.36	37.66 ± 0.36	0.14 ± 0.03	0.09 ± 0.02	0.02 ± 0.02	0.6 ± 0.03	0.00
IV	61.47 ± 0.25	38.04 ± 0.25	0.12 ± 0.02	0.04 ± 0.02	0.02 ± 0.01	0.30 ± 0.02	0.00
Control	58.93 ± 0.23	40.51 ± 0.23	0.17 ± 0.02	0.1 ± 0.02	0.02 ± 0.01	0.28 ± 0.01	0.00

*C, O, Na, Mg, Si, Ca, and Zr represent the elements carbon, oxygen, sodium, magnesium, silicon, calcium, and zirconium, respectively*.

#### Transmission Electron Microscopy Results

**Figure 6:**
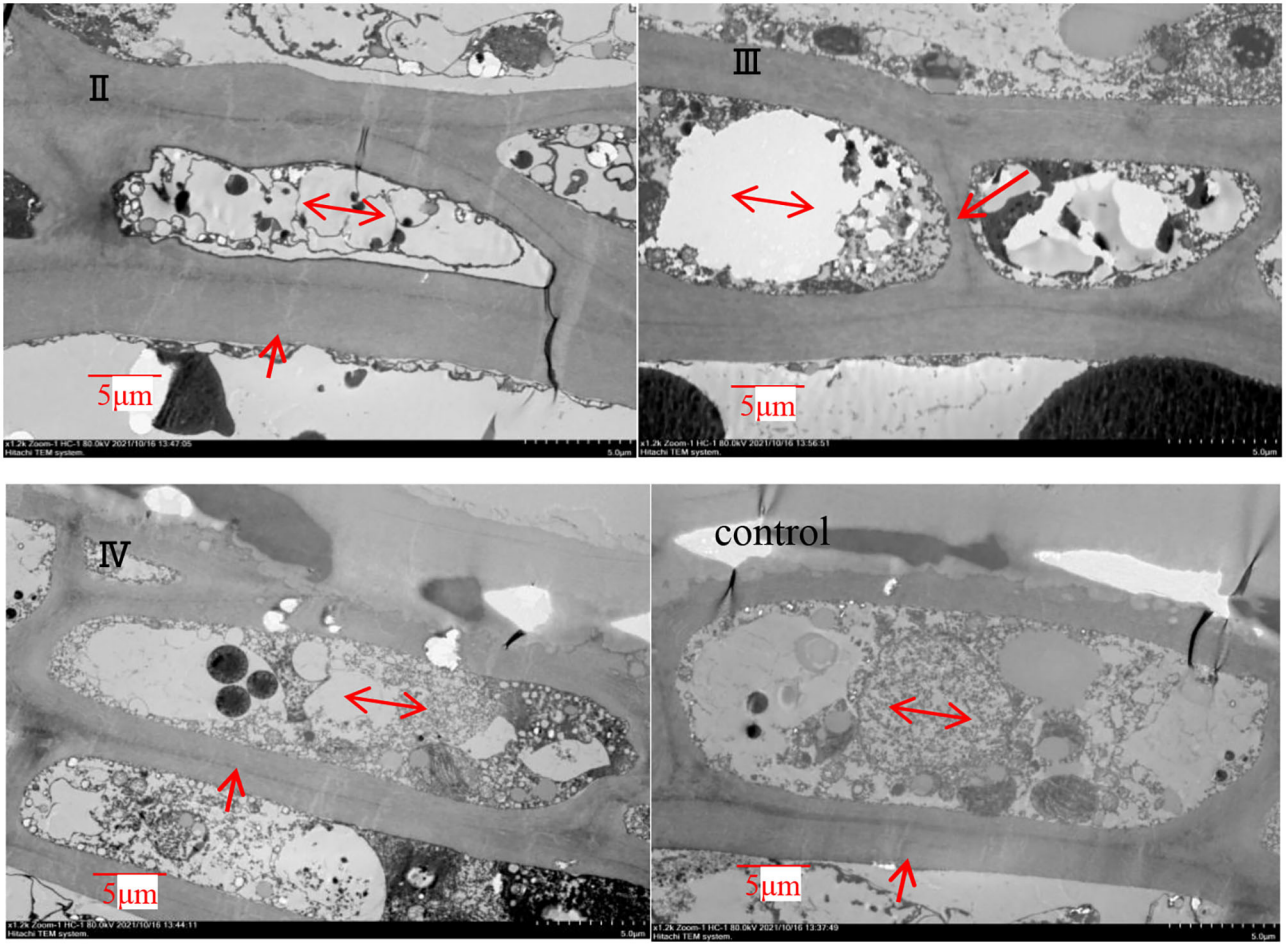
TEM of grape peels with calcium treatment in different stages (II represents ripening grape skins treated with calcium spray at flowering, III represents ripe grape skins treated with calcium spray during fruit development, and IV represents ripe grape skins treated with calcium spray at the end of fruit development) (cell wall, arrows; cell volume, double arrow).

**Figure 7:**
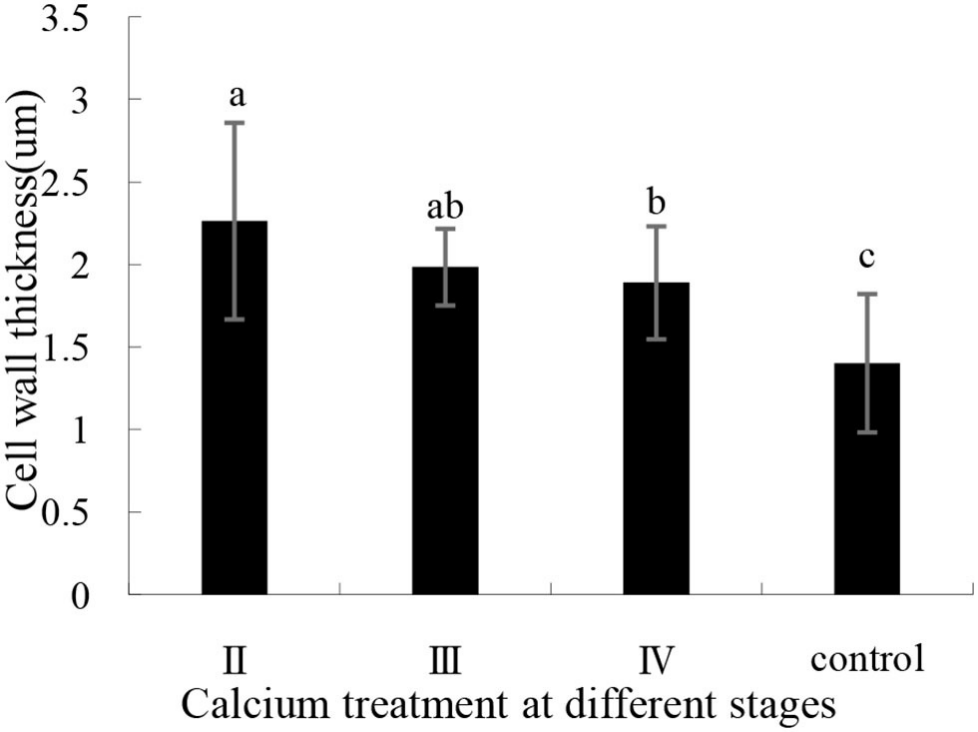
Pericarp cell wall thickness with calcium treatment in different stages. Data are presented as mean ± standard. Lowercase letters represent significant differences (*P* < 0.05).

The effects of calcium on grape peel cells are shown in Figure 6. Due to the poor effectiveness of (I), we did not conduct further studies on (I). The cell wall of the calcium treatment group was thicker (*P* ≤ 0.05) than that of the control group (Figure 6). Specifically, the II sample was increased by 61.35% compared with the control group (Figure 7), whereas the opposite pattern was observed for cell volume. The cell volume of the control group was significantly greater than that of the calcium treatment group. This study has shown that low Ca content in the pericarp is related to cell wall thickness, and that spraying calcium can increase cell wall thickness by increasing Ca content in the pericarp (Figure 7).

### Effects of Pre-Harvest Calcium Treatment on Grape Cracking in Different Storage Stages

**Figure 8:**
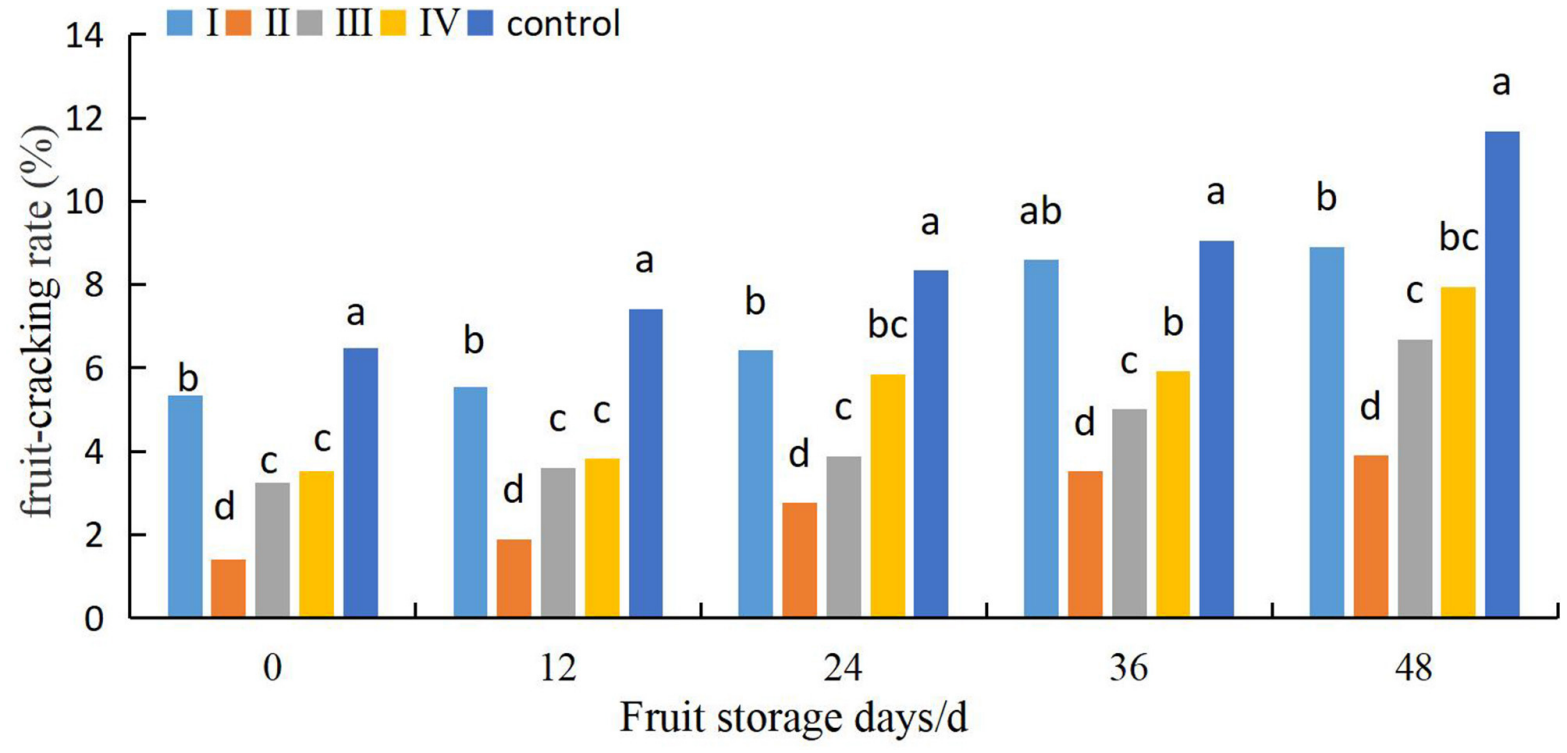
Effects of pre-harvest calcium treatment on post-harvest cracking of grapes. Data are presented as mean ± standard. Lowercase letters represent significant differences (*P* < 0.05).

The effects of different calcium treatments on fruit cracking in different storage stages are shown in Figure 8. The fruit cracking rate of control fruits was relatively low at the beginning of the storage period, increased rapidly, and was then maintained at relatively high levels. However, the fruits in the calcium treatment group maintained a lower rate of fruit cracking. Furthermore, when the fruits were stored for 48 days, the fruit cracking rate of the control fruits was 31.3, 200.2, 74.9, and 46.9% higher than that of the fruits of stages I, II, III, and IV, respectively. Meanwhile, the difference among the a II and I, III, and IV fruits was significant between 0 and 48 d of cold storage (*P* < 0.05).

## Discussion

Fruit cracking seriously affects the quality, post-harvest shelf life, and economic value of grapes. Therefore, it is very important to study the anti-cracking in grapes fruits. In this experiment, different periods of calcium treatment were used to explore the mechanism by which calcium prevents fruit cracking. Yu et al. (2020) found that exogenous calcium sprays had an anti-cracking effect on grapes when grapes were treated with calcium sprays 40 days after flowering. In our study, the calcium content of berry skins in the calcium treatment (Specially, calcium spray treatment during flowering) was significantly higher compared with that of the control. It may be because of the stronger calcium uptake during the flowering period compared to other periods in grapes (Davarpanah et al., 2018).

The fruit cracking rate and cracking index were represented about four and 10 times lower (*P* ≤ 0.05) in the flowering period sample (the best) than in the control group, respectively. It may be because the grapes absorb a lot of exogenous calcium, which leads to lower rate of cracking, while calcium spraying during the flowering stage has the best effect on calcium absorption (Yu et al., 2020). Meanwhile, the experimental results are similar to those of Deniz, Huang, and Bakeer. For example, Deniz (2014) found that calcium applications reduced the cracking index by 38–66% compared to cherries that did not receive foliar treatment, increasing the firmness of cherries by an average of 12%. In litchi, Huang et al. (2001) reported that contents of Ca in the cell wall as pectin-bound Ca in various parts of the pericarp were higher in cracking-resistant “Huaizhi” than in cracking-susceptible “Nuomici.” Bakeer (2016) confirmed that calcium chloride (1–2%) enhanced pomegranate vegetative growth parameters, yield, and fruit quality traits, and reduced fruit cracking, thereby improving the protection of the fruits from direct sunlight and the role of Ca in controlling physiological disorders of fruits.

ABA plays an important role in the grape ripening process. Previous studies have shown that ABA content in peels is related to fruit cracking (Koyama et al., 2010). In our study, the grape ABA content and related genes in samples treated with CaCl_2_ were significantly lower than those in the control samples (Figure 1). It is possible that calcium treatment of grapes reduced the expression of relevant transcription factors and key genes in the ABA metabolic pathway, thereby reducing ABA content, and the reduction in ABA content contributed to delayed fruit ripening (Yu et al., 2019). Meanwhile, the experimental results are similar to those of Yu et al. (2019). The rate of fruit cracking was reduced by reducing the content of endogenous ABA and the expression of related genes. In addition, Ca is closely related to anthocyanin synthesis. Anthocyanins have the ability to scavenge oxygen free radicals, and increase in anthocyanin content may be beneficial in delaying the aging of fruits (Blaga and Aleksandra, 2010). Ca may have reduced the amount of oxygen free radicals by reducing the amount of ABA in grapes, thus increasing the amount of anthocyanins (Martins et al., 2021b). In this study, the grape anthocyanin content and related genes were significantly higher in the samples treated with CaCl_2_ than in the control samples (Figure 4). The experimental results are similar to those of Peng et al. (2016), indicating that pre-harvest spray of CaCl_2_ enhanced anthocyanin accumulation in the strawberry fruit by stimulating the expression of anthocyanin structural genes including fruit-specific FvUGT1. Darzi and Ahsan (2010) found that the combination of 1.5 g/L calcium chloride and 0.5 g/L boric acid in the full bloom period can significantly reduce cherry fruit cracking and then significantly increase the anthocyanin content of the fruit.

Previous studies have shown that the composition of the cell wall in the peel influences the structure of the peel (Huang et al., 1999; Yang et al., 2016). Calcium may have reduced cell wall-related enzyme activity and expression of related genes by decreasing ABA content in grapes, thereby reducing water-soluble sugar content (Yu et al., 2019; Zhu et al., 2020). In this study, calcium is closely related to cell wall components, cell wall-related enzyme activity, and related genes (Figures 2, 3). The experimental results are similar to those of Michailidis and Yu. For example, Michailidis et al. (2017) reported that pre- and post-harvest Ca^2+^ applications increased total Ca^2+^ and cell wall-bound Ca^2+^ in apples, respectively, and that several color- and antioxidant-related genes were induced and reduced cracking. Yu et al. (2019) reported that calcium treatment reduced cell wall metabolism-related enzymatic activities and reduced WSP content, and reduced grape cracking. Moreover, the high content of SSs and TSSs in the fruit increases the osmotic pressure of the grape and increases the chance of fruit cracking (Considine and Kriedemann, 1972). In addition, in this study, pre-harvest calcium treatment has a good effect on post-harvest grape cracking (Figure 8). In previous studies, fruit storage was dominated by post-harvest calcium treatment. For example, Silva et al. (2012) found that “Isabel” grapes treated post-harvest with calcium chloride (doses5 and 2%) had decreased decay incidence, cracking, and mass loss. Stephane et al. (1994) showed that post-harvest spray of CaCl_2_ (doses 2 and 4%) increased calcium content and decreased fruit cracking in apples. However, pre-harvest calcium treatment usually has a better effect on preventing fruit cracking. Calcium uptake during fruit development is better than post-harvest calcium treatment. Ca sprays during berry development reduce the incidence of microcracks on fruit surface and lodging of filamentous fungi in these structures, reducing fruit decay at post-harvest (Martins et al., 2020, 2021a).

## Conclusions

The results of this study showed that calcium application decreased the cracking rate of grapes. Specifically, the best effect was obtained by spraying calcium in the flowering period. The decrease in fruit-cracking rate was attributed to the increased calcium content in the peels, which enhanced the peel stability and increased the anti-cracking fruit of grapes. Furthermore, the expression levels of glucose metabolism and ABA-related genes were reduced, which in turn decreased the PG, cellulase activities, and ABA content and delayed the degradation of pectin and cellulose. Moreover, the WSP, SS, and TSS contents were decreased, which increased fruit firmness and cell wall thickness, lowering peel turgor pressure and ultimately decreasing the susceptibility of fruits to cracking. In addition, calcium treatment reduced the post-harvest cracking rate of grapes.

## Data Availability Statement

The original contributions presented in the study are included in the article/supplementary material, further inquiries can be directed to the corresponding author/s.

## Author Contributions

HS and XZ: conceptualization. HS: methodology, data curation, and writing—original draft preparation. XH: formal analysis. XZ: writing—review and editing. WW and WZ: project administration. All authors have read and agreed to the published version of the manuscript.

## Funding

This study was supported by the National Undergraduate Innovation and Entrepreneurship Project (S202113809005), Ph.D. Introduction of Scientific Research Projects (2021HYBS03), and Natural Science Foundation of Hunan Province (Research on postharvest quality deterioration and regulation mechanism of Sunshine Rose grapes).

## Conflict of Interest

The authors declare that the research was conducted in the absence of any commercial or financial relationships that could be construed as a potential conflict of interest.

## Publisher's Note

All claims expressed in this article are solely those of the authors and do not necessarily represent those of their affiliated organizations, or those of the publisher, the editors and the reviewers. Any product that may be evaluated in this article, or claim that may be made by its manufacturer, is not guaranteed or endorsed by the publisher.
